# LED light sources improved the essential oil components and antioxidant activity of two genotypes of lemon balm (*Melissa officinalis* L.)

**DOI:** 10.1186/s40529-021-00316-7

**Published:** 2021-06-05

**Authors:** Tayebeh Ahmadi, Leila Shabani, Mohammad R. Sabzalian

**Affiliations:** 1 Department of Plant Science, Faculty of Science, Shahrekord University, Shahrekord, Iran; 2 Research Institute of Biotechnology, Shahrekord University, Shahrekord, Iran; 3 Department of Agronomy and Plant Breeding, College of Agriculture, Isfahan University of Technology, 84156-83111 Isfahan, Iran

**Keywords:** Antioxidant activity, Essential oil, Light-emitting diodes (LEDs), *Melissa officinalis*

## Abstract

**Background:**

Nowadays, light-emitting diodes (LEDs) as a new lighting technology, have been emerged as an alternative source of light for plants due to their wavelength specificity, the narrow width of their bands, small size, solid structure, long lifetime, and low heat generation. Here we investigated the effect of different LED light sources on the essential oil components and antioxidant activity of *Melissa officinalis*. Two genotypes of lemon balm (Ilam and Isfahan) were subjected to four artificial light treatments, including white, red, blue, red + blue LEDs, and greenhouse light as natural lighting.

**Results:**

The LED lights significantly increased shoot fresh and dry weights and leaf number in the two genotypes as compared to greenhouse condition. The results showed that the content and composition of essential oil in the two genotypes were variable under different light treatments and the total amount of compounds in the Ilam genotype was higher than the other genotype. The results of analysis of the essential oil by GC/MS indicated that the highest amount of monoterpenes in the genotypes was related to citronellal under red + blue LED lamps (15.3 and 17.2% in Ilam and Isfahan genotypes, respectively) but blue, white, and greenhouse condition had the most effect on sesquiterpenes content in both genotypes. The results showed that the observed variation between the two genotypes in the essentials oil composition was related to the relative percentage of the constituents and not to the appearance or lack of a specific component. Red + blue lighting also provided the highest radical scavenging activity in both genotypes (80.77 and 82.09% for Ilam and Isfahan genotypes, respectively). Based on principal component analyses (PCA), three main groups were identified regarding genotypes and all light treatments.

**Conclusions:**

Overall, results indicated that the essentials oil composition of two genotypes of lemon balm was affected both qualitatively and quantitatively by different LED light sources; hence, LED lights might be used to improve monoterpenes, sesquiterpenes, and antioxidant activity in the selected genotypes.

**Supplementary Information:**

The online version contains supplementary material available at 10.1186/s40529-021-00316-7.

## Introduction

*Melissa officinalis* (lemon balm) is native to the north of Iran, western Asia, southern Europe, and northern Africa (Noorul Basar and Zaman 2013). This medicinal plant belongs to the family Lamiaceae that whose scented leaves have been consumed as a spice for salads and cold drinks, decoction, infusion, or directly used in food for decades. Several studies have shown antioxidant, hypoglycemic, hypolipidemic, anti-cancer, anti-depressant, sedative, and anti-inflammatory effects, in addition to known effects of this plant for digestive problems, rheumatism, or headache (Weidner et al. 2015; Birdane et al. 2007). Generally, the essential oil extracted from the aerial parts of lemon balm (0.02–0.8%, with the main components being citral and citronellal) is one of the classes of compounds that are useful for food, nutrition, pharmacological and cosmetic applications, and antioxidant activities of the plant (Mazzanti et al. 2008).

Many secondary metabolites, such as flavonoids, essential oils, and phenolic acids are produced in response to environmental stresses (Weitzel and Peterson 2010). light, As an important environmental factor, is an essential source of energy for the photosynthesis of a plant, as well as an important message for plant growth and development. Nowadays, new technologies such as light-emitting diodes (LEDs) lamps provide better growth of plants by emitting an appropriate intensity and wavelength of light as an alternative source for sunlight (Sabzalian et al. 2014). LEDs can also stimulate the generation of secondary metabolites and essential oils in plants (Ghaffari et al. 2019; Tohidi et al. 2019). Blue LED light made the highest amount of essential oil in basil leaves (Amaki et al. 2011) and *Perovskia atriplicifolia* (Gaffari et al. 2019). The highest thymol content was observed in *Thymus migricus* under blue LED light (Tohidi et al. 2019). Red LED light significantly increased lutein and glycosinolate in *Brassica oleracea* leaf (Lefsrud et al. 2008) and phenylacetaldehyde in *Petunia hybrid* flower (Colquhoun et al. 2013). Sabzalian and co-workers (2014) recorded a four-fold increase in essential oil production of *Mentha longifolia* under red + blue LED light. The positive effect of blue, white, and green LED lights was equally reported by Jung et al. (2013) on the antioxidant activity of rice leaves. Johkan and his colleagues (2010) also stated that the antioxidant activity of lettuce seedlings treated with blue LEDs was higher than that of red and fluorescent lights. Additionally, the variability among plant genotypes influences the quantity and quality of secondary metabolites, which leads to great differences in secondary metabolite synthesis (Gahler et al. 2003). Several studies have revealed that the essential oil components and yield can vary with plant genotypes (Rajendra et al. 2016; Gholami-Zali and Ehsanzadeh 2018). However, little data have been published about the variation in the essential oil composition between the lemon balm genotypes.

Today, the global strategy in the production of medicinal and aromatic plants is to improve the quantity, quality, and health of their essential oils, and due to the emergence of adverse effects of chemicals used in agriculture, such as fertilizers and pesticides, the tendency to use approaches that are healthier and more environmentally friendly is on the rise. In this regard, the use of LED lamps technology while providing plants with better growth and production, also helps to better protect the environment. The goal of this study was to investigate the effects of four light wavelengths of LED lamps on two genotypes of lemon balm (Ilam and Isfahan), regarding growth, antioxidant activity, and essential oil constituents.

## Material and methods

### Plant material

The plant material of this study was *Melissa officinalis* plants that were collected from fields in Eyvan city of Ilam province (voucher no. 4897, with geographical coordinates 33°53′N 46°11′E) and Isfahan city of Isfahan province (voucher no. 4898, with geographical coordinates 32°38′41″N 51°40′03″E). Three rhizomes were planted in each pot (15 cm diameter) containing 3:1 loam-sandy soil (30 pots for two genotypes).

### System of light and experimental condition

The experiment was conducted in two separate environments including greenhouse and LED incubators according to a completely randomized design. On March 11, 2016, 24 pots (12 from each genotype) were incubated in four growth cabinets (i.e. 3 pots from each genotype per each cabinet). Each cabinet’s lighting system contained red (660 nm), blue (460 nm), white (380–760 nm) and red + blue (70:30 ratio) LED lamps with a light intensity of 300 μmol.m^−2^.s^−1^, with a 16 h illumination and 8 h darkness and at 25 ± 2 °C. On the same date, 6 pots (3 pots from each genotype) were kept in the research greenhouse of Isfahan University of Technology, in similar conditions under natural sunlight. All pots were irrigated daily with water and once a week with 1/2 Hoagland solution. After 7 weeks, on April 29, plants were transferred to the laboratory for analysis.

### Assessment of growth parameters

First, the samples were washed with distilled water and their excess moisture was removed with filter paper. The fresh weight of plant shoots was determined in grams after cutting the roots from the crown. To calculate the dry weight, each sample, after being placed in paper bags, was dried in an oven at 65 °C for 48 h and then weighed in grams. The leaves were counted separately in each pot.

### Extraction of essential oil

Seven weeks after exposure to LED lighting, 10 g of dry leaves from each light treatment was used for essential oil extraction. The dried and ground leaves were submitted to 150 ml water distillation using a Clevenger apparatus. The sample of oils was extracted in triplicate. The essential oil was extracted for 6 h at 70–80 °C temperature. The volatile fraction collected was kept in sealed glass vials and stored at 4 °C before GC–MS analysis. The essential oil content (%) was calculated as follow:

$${\text{Essential oil content }}\left( \% \right)\, = \,{\text{Mass of isolated essential oil }}\left( {\text{g}} \right)/{\text{dried shoot }}\left( {\text{g}} \right)\, \times \,{1}00$$

### Gas chromatography-mass spectrometry (GC–MS)

Gas chromatography-mass spectroscopy (GC–MS) analysis for the essential oil composition was carried out on a Hewlett–Packard 5890 gas chromatograph (Hewlett–Packard, EquipNet, Inc. 50 Hudson Road Canton, MA 02021 USA) coupled to an HP 5970 mass-selective detector (MSD) (Hewlett–Packard, EquipNet, Inc. 50 Hudson Road Canton, MA 02021 USA) using a fused silica ultra-performance cross-linked methyl silicone column (50 mm length, 0.2 mm inner diameter, film thickness 25 µm). Temperature programming was done from 100 to 250 °C at a rate of 4 °C min^−1^. Helium was used as the carrier gas at a 1 ml min^−1^ flow rate. Ionization energy at 70 eV and 250 °C temperature were ordered for the ion source (Li et al. 2009). Identification of the essential oil compounds was based on the comparison of their spectral fragmentation with the data reported in the NIST 08 (National Institute of Standards and Technology), Mass Spectra Libraries, and those reported in Davies 1990 (Additional files 1, 2, 3, 4). Retention indices (RI) were determined with C5–C26 alkane standards as reference. They were confirmed by a nonpolar HP-5MS column through comparison of their mass spectra against those recorded in the NIST 08, Willey 275.L (ChemStation data system), and those reported in the literature (Davies, 1990).

### Determination of antioxidant activity

#### 2,2-diphenyl-1-picrylhydrazyl radical scavenging activity

The radical scavenging activity (RSA) of the extracts was monitored using the stable free radical 2,2-diphenyl-1-picrylhydrazyl (DPPH) following the method described by Kulisica et al. (2004). Extract solutions (1 ml) were mixed with 1 ml of a freshly prepared DPPH solution (0.1 mM in methanol) and 3 ml of 96% ethanol. The mixture was shaken vigorously and left to stand at room temperature for 30 min in dark (until stable absorbance values were obtained). The reduction in the DPPH radical content was measured by monitoring the decrease in absorption at 517 nm. RSA of the extracts was calculated by the following formula:

$$\% {\text{ Radical scavenging activity}}\, = \,({\text{control OD }}\left( {\text{optical density}} \right){-\!\!-}{\text{sample OD}}/{\text{control OD}})\, \times \,{1}00.$$

Methanol (80%) and DPPH solution (0.1 mM, 5 ml) were used separately as a blank and control sample, respectively. The test also was done in triplicate.

## Statistical analysis

Since the cultivation of plants was in greenhouse and 4 incubators in the laboratory, considered as an experiment in separate environments, so for the measured factors, the significance of variation was assessed using the combined analysis of data using SAS statistical program (ver. 8; SAS Institute Inc., Cray, NC, USA) and the least significant differences method was used for mean comparisons. Identification of interrelationships between the genotypes of *Melissa officinalis* subjected to LED lighting with measured traits was done using principal component analysis (PCA).

## Results

### Growth parameters

The effect of different light sources and genotypes on growth parameters was significant (Table 1). Table 2 showed that the highest shoot fresh and dry weights and leaf number were observed under the red + blue LED light source in plants of both genotypes.

**Table 1:** Analysis of variance (mean of squares) of genotype, light, and their interactions on growth factors and major compounds of essential oil in *Melissa officinalis*

Source of variation	df	Shoot fresh weight (g)	Shoot dry weight (g)	Leaf number	γ-3-carene	Linalool	Trans-Carveol	Citronellal	Citronellol	Citral	ß-Caryophyllene	Caryophyllen epoxide	Germacrene-D
Light	4	596.7^**^	14.3714.37^**^	75676.36^**^	7.144^**^	11.787^**^	15.9^**^	28.792^**^	17.412^**^	9.072^**^	9.072^**^	9.072**	9.072^**^
Rep (light)	10	6.69	0.65	884.54	0.009	0.12	0.12	0.12	0.008	0.012	0.012	0.012	0.012
Genotype	1	487.14^**^	5.61^**^	25317.07^*^	7.203^**^	0.300^**^	7.203^*^	12.675^**^	5.808^**^	0.192^**^	0.192^**^	0.192^**^	0.192^**^
Light*Genotype	4	50.97^**^	0.71^**^	22707.42^**^	2.665^**^	1.860^**^	0.798^**^	1.057^**^	1.698^**^	1.137^**^	1.137^**^	1.137^**^	1.137^**^
Error	10	2.83	0.32	876.4	0.017	0.008	0.008	0.008	0.012	0.008	0.008	0.008	0.008

*, **significant variation at p ≤ 0.05 and p ≤ 0.01, respectively

**Table 2:** Interaction of genotype and light on growth indices in two lemon balm genotypes under different levels of light (Data in the table represent means ± SE and different letters indicate a significant difference at the probability level of 0.05 based on LSD test)

Light/Genotype	Shoot fresh weight (g)		Shoot dry weight (g)			Leaf number	
	Ilam	Isfahan	Ilam	Isfahan		Ilam	Isfahan
White LED	29.65 ± 2.55^b^	25.46 ± 0.04^c^	4.7 ± 0.2^c^	3.74 ± 0.1^e^		387 ± 17^d^	317 ± 17^e^
Red + Blue LED	44.32 ± 1.1^a^	45.11 ± 0.66^a^	7.17 ± 0.4^a^	7.74 ± 0.2^a^		646 ± 20^a^	688 ± 15^a^
Red LED	32.6 ± 0.5^b^	42.26 ± 2.54^a^	5.53 ± 0.1^c^	6.4 ± 0.3^b^		556 ± 50^b^	544 ± 6^b^
Blue LED	30 ± 3.97^b^	22.25 ± 0.12^d^	4.70 ± 0.1^d^	3.39 ± 0.04^f^		253 ± 22^e^	330 ± 49^e^
Greenhouse	22.82 ± 1.04^d^	18.4 ± 0.63^e^	3.54 ± 0.4^e^	3.54 ± 0.1^e^		469 ± 12^c^	213 ± 40^f^

### Essential oil content and constituents

The essential oil content of two genotypes of *M. officinalis* was not significantly affected by different LED light sources when compared to the greenhouse condition (in the range of 0.27–0.32%, data not shown). However, the effect of different light sources and genotypes on major compounds of lemon balm essential oil was significant (Table 1). A total of thirty-eight compounds were identified using GC–MS analysis (Table 3). A considerable difference was observed in the concentration of essential oil constituents in both genotypes of *M. officinalis* grown under various light sources (Table 3). In plants of Ilam genotype, ß-cubebene and citronellal had the lowest and highest amounts among all of the identified constituents under red and red + blue LEDs, respectively with 0.6 and 15.3%, but in the other genotype, the lowest amount of essential oil compounds belonged to bicyclo[2.2.1]heptan-2-one, ß-ionone, and eugenol in plants that were subjected to red, blue, and red + blue LEDs (0.8%). Among essential oil compounds, citronellal had the highest content (17.2%) under red + blue LED lights in the Isfahan genotype.

**Table 3:** Essential oil composition in two genotypes of *Melissa officinalis* L. under various light treatments. Data in the table represent means ± SE and different letters indicate a significant difference at the probability level of 0.05 based on LSD test

Compounds	Chemical formula	RI (min)	Ilam genotype					Isfahan genotype				
			White	Red + Blue	Red	Blue	Greenhouse	White	Red + Blue	Red	Blue	Greenhouse
1,3- octadiene	C8H14	7.6	0.8 ± 0.1^b^	-	1.5 ± 0.1^a^	1.3 ± 0.1^a^	1 ± 0.05^b^	1 ± 0.1^b^	1.2 ± 0.05^a^	1 ± 0.1^b^	-	0.7 ± 0.05^c^
Alpha-pinene	C10H16	9.8	2.8 ± 0.2^a﻿^	2 ± 0.1^c^	1.9 ± 0.1^c^	2.2 ± 0.2^c^	3.1 ± 0.3^a^	1.7 ± 0.1^d^	2 ± 0.1^c^	1.7 ± 0.1^d^	2.6 ± 0.1^b^	2.4 ± 0.1^b^
1,Octen-3-ol	C8H16O	10.5	1.1 ± 0.3^a﻿^	1.2 ± 0.2^a﻿^	1.1 ± 0.1^a^	1.4 ± 0.3^a^	0.9 ± 0.1^a^	0.8 ± 0.3^a^	1.1 ± 0.1^a^	0.9 ± 0.05^a^	1.1 ± 0.2^a^	1.3 ± 0.1^a^
Myrcene	C10H16	11	2 ± 0.2^a^	0.9 ± 0.1^c﻿^	1.1 ± 0.1^c^	1.4 ± 0.07^b^	1.9 ± 0.1^a﻿^	1.3 ± 0.1^b^	1.2 ± 0.05^c^	1.5 ± 0.05^b^	1.8 ± 0.1^a^	1.3 ± 0.1^c^
γ-3-Carene	C10H16	11.3	6.7 ± 0.2^d^	4.9 ± 0.1 ^h^	6 ± 0.3^e^	8 ± 0.1^b^	8.5 ± 0.2^a^	7.4 ± 0.1^c^	4.5 ± 0.1^i^	5.4 ± 0.1 ^g^	6.1 ± 0.07^e^	5.8 ± 0.2^f^
Alpha phellandrene	C10H16	11.8	1.3 ± 0.3^d^	2 ± 0.05^b^	2.6 ± 0.3^a^	2.1 ± 0.1^b^	1.9 ± 0.1^b^	1.3 ± 0.2^d^	2 ± 0.2^b^	1.5 ± 0.05^c^	1.7 ± 0.3^c^	2.1 ± 0.1^b^
Para cymene	C10H14	12.2	1 ± 0.05^c^	1.5 ± 0.2^b^	2 ± 0.05^a^	1.6 ± 0.1^b^	1.2 ± 0.2^c^	1.3 ± 0.3^c^	2 ± 0.07^a^	1.5 ± 0.2^b^	1.7 ± 0.2^b^	2.1 ± 0.1^a^
Limonene	C10H16	12.6	2.8 ± 0.2^a^	2.2 ± 0.2^b^	1.9 ± 0.07^c^	2.3 ± 0.1^b^	3 ± 0.3^a^	1.4 ± 0.05^d^	1.2 ± 0.07^d^	2.1 ± 0.1^b^	2.6 ± 0.1^a^	2.3 ± 0.05^b^
Eucalyptol	C10H18O	12.9	1.7 ± 0.2^c^	1.3 ± 0.2^d^	2.1 ± 0.1^a^	1.7 ± 0.2^c^	1.3 ± 0.4^d^	2.2 ± 0.07^a^	1.2 ± 0.4^d^	1.6 ± 0.2^c^	2 ± 0.1^a^	1.9 ± 0.3^b^
1,3,6,-Octatriene	C8H12	13.5	0.9 ± 0.2^e^	1.2 ± 0.3^c^	1.3 ± 0.1^b^	1.4 ± 0.2^b^	1.7 ± 0.05^a^	1 ± 0.2^d^	1 ± 0.3^d^	1.3 ± 0.1^b^	1.1 ± 0.2^c^	0.7 ± 0.1^e^
γ-Terpinene	C10H16	14.1	2.5 ± 0.05^a^	1.8 ± 0.3^d^	2.4 ± 0.1^a﻿^	1.5 ± 0.4^e﻿^	2 ± 0.2^c^	1.4 ± 0.4^e﻿^	2.5 ± 0.1^a^	2.2 ± 0.2^b^	1.9 ± 0.3^d^	2.5 ± 0.05^a^
Linalool	C10H18O	14.4	6.5 ± 0.2^e^	8.9 ± 0.2^a^	7.1 ± 0.3^d^	5.3 ± 0.2 ^g﻿^	4.6 ± 0.3 ^h﻿^	6 ± 0.2^f^	8 ± 0.4^b^	7.4 ± 0.3^c^	7.3 ± 0.3^c﻿^	4.7 ± 0.4 ^h^
Cis-Sabinene	C10H16	14.8	2.3 ± 0.1^a^	1.5 ± 0.4^c﻿^	2 ± 0.1^a﻿^	1.6 ± 0.3^c﻿^	1.8 ± 0.3^c﻿^	1.9 ± 0.2^b^	2 ± 0.2^a^	2.3 ± 0.07^a^	1.7 ± 0.2^c^	2.2 ± 0.1^a^
3-methyl-2(methyl-2-2butenyl)	C10H16O2	15.7	0.9 ± 0.2^d﻿^	1.2 ± 0.07^c^	0.8 ± 0.1^d﻿^	1.1 ± 0.05^c^	1.5 ± 0.2^c^	0.8 ± 0.1^d^	0.9 ± 0.2^d^	1.1 ± 0.05^c﻿^	1.4 ± 0.2^b^	2.2 ± 0.2^a^
β-Thujone	C10H16O	16.3	2.5 ± 0.2^b^	1.7 ± 0.05^e﻿^	2.2 ± 0.2^c﻿^	1.8 ± 0.2^d﻿^	3 ± 0.2^a^	1.3 ± 0.3^f﻿^	1.3 ± 0.2^f^	1.9 ± 0.1^d^	2.3 ± 0.3^c^	1.8 ± 0.2^d^
5-Hepten-1-ol	C7H14O	17.4	1.2 ± 0.1^c﻿^	1.3 ± 0.07^c﻿^	1.6 ± 0.05b	2 ± 0.05^a﻿^	1.8 ± 0.2^a﻿^	1.8 ± 0.1^a^	0.8 ± 0.07^e^	1.2 ± 0.1^c^	1 ± 0.1^d^	1.4 ± 0.3^b^
Bicyclo[2.2.1]heptan-2-one	C7H10O	17.8	1.5 ± 0.2^a﻿^	1 ± 0.3^c﻿^	1.2 ± 0.1^b﻿^	1.4 ± 0.2^a﻿^	1.1 ± 0.2^b^	1.6 ± 0.2^a^	1.1 ± 0.2^b^	0.8 ± 0.2^c^	0.9 ± 0.1^c^	1.1 ± 0.1^b﻿^
Isopulegol	C10H18O	18.2	1.9 ± 0.1^c﻿^	1.4 ± 0.2^d﻿^	2.1 ± 0.2^b﻿^	1.5 ± 0.1^d^	2.6 ± 0.2^a^	2.3 ± 0.3^b﻿^	2 ± 0.3^b﻿^	1.6 ± 0.1^d﻿^	2.1 ± 0.2^b﻿^	2.7 ± 0.2^a﻿^
Trans-carveol	C10H16O	19.5	7.6 ± 0.2^f﻿^	10.2 ± 0.3^b﻿^	8.5 ± 0.3^﻿e^	6.8 ± 0.2^h^	5.7 ± 0.4^i^	7.5 ± 0.3^f﻿^	11.1 ± 0.3^a﻿^	9.4 ± 0.1^c﻿^	8.7 ± 0.3^d﻿^	7 ± 0.1 ^g﻿^
Citronellal	C10H18O	20.1	11.8 ± 0.2^g﻿^	15.3 ± 0.2^b﻿^	13.4 ± 0.3^e^	11.3 ± 0.4^h^	10.2 ± 0.4^j^	12.7 ± 0.3^f﻿^	17.2 ± 0.3^a﻿^	14.4 ± 0.4^c﻿^	13.7 ± 0.7^d﻿^	10.5 ± 0.5^i﻿^
Isoborneol	C10H18O	20.4	1.8 ± 0.05^b﻿^	1.5 ± 0.1^c^	1.1 ± 0.1^d^	1.7 ± 0.05^b^	2.2 ± 0.2^a^	2 ± 0.05^a﻿^	1.7 ± 0.1^b﻿^	2.1 ± 0.1^a﻿^	1.8 ± 0.07^b﻿^	2.3 ± 0.1^a﻿^
Citronellol	C10H20O	21.8	9.7 ± 0.5^e^	7.3 ± 0.4^i﻿^	9.1 ± 0.4^f^	11.2 ± 0.3^b^	11.8 ± 0.2^a^	10.3 ± 0.5^d﻿^	6.5 ± 0.4^j﻿^	8.1 ± 0.3 ^h﻿^	8.8 ± 0.3 ^g﻿^	11 ± 0.4^c^
1,3,8,-Ρ-menthatriene	C10H14	22.4	1.6 ± 0.05^c^	1.8 ± 0.1^b^	1.4 ± 0.07^d^	1.5 ± 0.1^c﻿^	1.9 ± 0.1^b^	1.3 ± 0.1^e^	1.3 ± 0.1^e^	2 ± 0.07^a^	1.7 ± 0.3^b﻿^	2.1 ± 0.07^a﻿^
Citral	C10H16O	23.9	6.4 ± 0.4^e^	8.2 ± 0.3^b^	7.4 ± 0.3^c^	5.5 ± 0.07^h^	5.7 ± 0.4^g^	5.9 ± 0.05^f^	9.1 ± 0.5^a^	6.9 ± 0.3^d^	6.8 ± 0.3^d^	5.3 ± 0.1^i﻿^
3,6,-Octadienoic acid	C10H16O2	24.2	0.8 ± 0.05^f﻿^	0.9 ± 0.05^e^	1 ± 0.1^d^	1.1 ± 0.1^c^	1.2 ± 0.07^﻿b^	-	1.1 ± 0.1^b﻿^	1.7 ± 0.2^a﻿^	1 ± 0.1^d^	1.2 ± 0.05^b^
Thymol	C10H14O	25.1	2.8 ± 0.1^b﻿^	2 ± 0.07^f^	2.3 ± 0.1^e﻿^	3 ± 0.1^a﻿^	2.4 ± 0.1^d﻿^	2.6 ± 0.1^c^	1 ± 0.1^i^	1.7 ± 0.1 ^g﻿^	1.2 ± 0.1^h﻿^	1.7 ± 0.1 ^g^
Carvacrol	C10H14O	25.5	1.4 ± 0.05^f^	1.3 ± 0.1^f﻿^	1.9 ± 0.2^e﻿^	2.2 ± 0.2^c﻿^	1.7 ± 0.07^a﻿^	2.6 ± 0.1^a^	1.9 ± 0.05^e^	2.6 ± 0.1^a^	2.1 ± 0.07^d﻿^	2.3 ± 0.2^b﻿^
Carvacrol acetate	C12H16O2	26	2.6 ± 0.2^a^	1.8 ± 0.05^c﻿^	2.2 ± 0.2^b﻿^	2.5 ± 0.2^a﻿^	1.3 ± 0.1^e﻿^	1.6 ± 0.07^d^	2.1 ± 0.3^b^	1.9 ± 0.1^c^	1.6 ± 0.05^d﻿^	2.3 ± 0.1^b^
ß-Caryophyllene	C15H24	29.8	1 ± 0.05^i^	0.9 ± 0.05^j^	1.1 ± 0.07^h^	1.8 ± 0.1^d﻿^	1.4 ± 0.05^f^	1.6 ± 0.1^e^	2.1 ± 0.1^b^	1.9 ± 0.07^c^	1.6 ± 0.05^e^	2.3 ± 0.2^a^
Caryophyllen epoxide	C15H24O	30.3	1.8 ± 0.1^c^	1.9 ± 0.1^c^	1.7 ± 0.05^d^	1.5 ± 0.1^e﻿^	1.2 ± 0.2^f﻿^	2.1 ± 0.07^b^	1.2 ± 0.2^f^	1.7 ± 0.1^d^	1.1 ± 0.05^f^	2.3 ± 0.1^a﻿^
Calamenene	C15H22	30.7	0.9 ± 0.1^f^	1.5 ± 0.2^b﻿^	1 ± 0.05^e^	1.2 ± 0.2^d^	1.5 ± 0.2^b﻿^	1.4 ± 0.1^c^	1.3 ± 0.1^d^	1.5 ± 0.2^b﻿^	1.2 ± 0.2^d^	1.8 ± 0.1^a^
α-Humulene	C15H24	31.7	1 ± 0.07^d^	1.3 ± 0.1^b^	0.9 ± 0.1^e^	1.1 ± 0.2^c^	0.8 ± 0.05^f﻿^	1.5 ± 0.1^a^	1.2 ± 0.07^c^	1 ± 0.1^d^	1.3 ± 0.1^b^	0.9 ± 0.05^a﻿^
Germacrene-D	C15H24	32.9	1.6 ± 0.2^c^	1.5 ± 0.2^c^	2 ± 0.07^b^	1.7 ± 0.1^c^	1.2 ± 0.1^d﻿^	2.2 ± 0.05^a^	1 ± 0.1^e^	1.2 ± 0.1^d^	1.5 ± 0.07^c^	1.3 ± 0.1^d^
ß-Ionone	C13H20O	33.1	1 ± 0.05^c^	1.8 ± 0.2^a^	1.3 ± 0.2^b﻿^	1 ± 0.1^c﻿^	0.7 ± 0.05^d^	1.4 ± 0.2^b^	1.3 ± 0.1^a^	1.1 ± 0.05^c^	0.8 ± 0.1^d^	1 ± 0.2^c^
ß-bisabolene	C15H24	34	0.9 ± 0.05^d^	1 ± 0.1^c^	1.2 ± 0.1^b^	1.5 ± 0.07^a^	1.2 ± 0.07^b^	0.8 ± 0.1^d^	1.1 ± 0.07^c^	0.9 ± 0.1^d^	1 ± 0.05^c^	1.4 ± 0.2^a^
Eugenol	C10H12O2	35.1	1.1 ± 0.05^b^	0.9 ± 0.1^c^	-	0.8 ± 0.05^c^	1 ± 0.07^b^	1.1 ± 0.05^b^	0.8 ± 0.1^c^	1.2 ± 0.1^a^	0.9 ± 0.1^c^	1.3 ± 0.07^a^
α-Muurolene	C15H24	36.6	0.8 ± 0.1^d﻿^	0.8 ± 0.07^d^	1 ± 0.1^c^	1.1 ± 0.05^c^	1.3 ± 0.2^a﻿^	1.2 ± 0.1^b^	1.2 ± 0.07^b^	1.4 ± 0.1^a^	1 ± 0.07^c^	1.2 ± 0.1^b^
ß-Cubebene	C15H24	40.2	1.4 ± 0.07^a^	1.4 ± 0.1^a^	0.6 ± 0.1^d^	1.3 ± 0.1^a^	1 ± 0.05^c﻿^	1.4 ± 0.1^a^	1 ± 0.1^c^	1.4 ± 0.05^a^	1 ± 0.1^c^	1.2 ± 0.07^b^
Monoterpenes	____	____	79.7	81.9	80.7	76.7	76.8	76	80.6	80.2	78.8	78.5
Sesquiterpenes	____	____	10.4	11.1	10.8	12.2	10.3	13.6	11.4	10.3	10.5	13.4
Others	____	____	8.3	6.3	8.5	10.5	10.2	8.1	8	9.2	7.4	7.7
The total amount of compounds			98.4	99.3	100	99.4	97.3	97.7	100	99.7	96.5	99.6
Number of compounds			38	37	37	38	38	37	38	38	37	38

#### Monoterpenes content under various light treatments in two genotypes of *M. officinalis*

The number of identified monoterpenes in the two genotypes of lemon balm was 21 compounds. The amount of these compounds was different under various light treatments. In the Ilam genotype, the highest amount of monoterpenes was observed in incubators with white, red + blue, and red LEDs, but in the Isfahan genotype, these compounds were higher under blue LED and greenhouse condition. The red + blue LED light treatment in both genotypes produced the highest amount of monoterpene compounds compared to the greenhouse condition (Table 3).

In the Ilam genotype, monoterpene compounds ranged from 0.9% for myrcene under red + blue LED to 15.3% for citronellal under the same light treatment (Table 3). Under LED light treatments, monoterpenes such as myrcene, alpha-phellandrene, para cymene, eucalyptol, γ-terpinene, linalool, cis-sabinene, trans-carveol, citronellal, citral, thymol, carveol, and carveol acetate were more affected than greenhouse condition. In this genotype, myrcene (0.9%) and para cymene (1%) had the lowest content among identified monoterpenes, regardless of light treatment. In this genotype, the red + blue LED treatment resulted in the highest amount of major essential oils namely citronellal (15.3%), trans-carveol (10.2%), linalool (8.9%), and citral (8.2%) compared to the other lights.

The range of monoterpene composition in the Isfahan genotype changed from 1% for thymol under white LED light to citronellal with 17.2% under red + blue LED light (Table 3). Myrcene, limonene, eucalyptol (1.2%), and thymol (1%) had the lowest values among known monoterpenes, regardless of light treatments. Alpha-pinene, myrcene, γ-3-carene, limonene, eucalyptol, linalool, cis-sabinene, β-thujone, trans-carveole, citronellal, citral, thymol, and carveol had the most contents in plants grown in incubators with LED lamps compared to the greenhouse condition. Among light treatments, red + blue LED light had the highest contribution to increasing the amount of major essential oils including citronellal (17.2%), trans-carveole (11.1%), citral (9.1%), and linalool (8.9%).

#### Sesquiterpenes content under various light treatments in two genotypes of *M. officinalis*

According to the results shown in Table 3, the number of sesquiterpene compounds in lemon balm genotypes was also significantly affected by light treatments. In Ilam and Isfahan genotypes, the highest amount of these compounds were obtained under blue and white LEDs and greenhouse conditions, respectively. In the Ilam genotype, LED light treatments increased the level of sesquiterpenes such as ß-caryophyllene, α-humulene, germacrene-D, ß-ionone, and ß-cubebene but decreased the amount of caryophyllene epoxide, calamenene, and α-muurolene compared to the greenhouse condition. In contrast, in the Isfahan genotype, only α-humulene, ß-ionoe, and germacrene-D levels were higher in most LED treatments compared to greenhouse conditions. The reduction in the amount of ß-caryophyllene, caryophyllene epoxide, and calamenene treated with LED light was significantly higher than the greenhouse condition. In plants of two genotypes, caryophyllene epoxide, germacrene-D, and ß-caryophyllene were sesquiterpene compounds with the highest amount, respectively, regardless of light treatments.

### Radical scavenging activity analysis

**Fig. 1:**
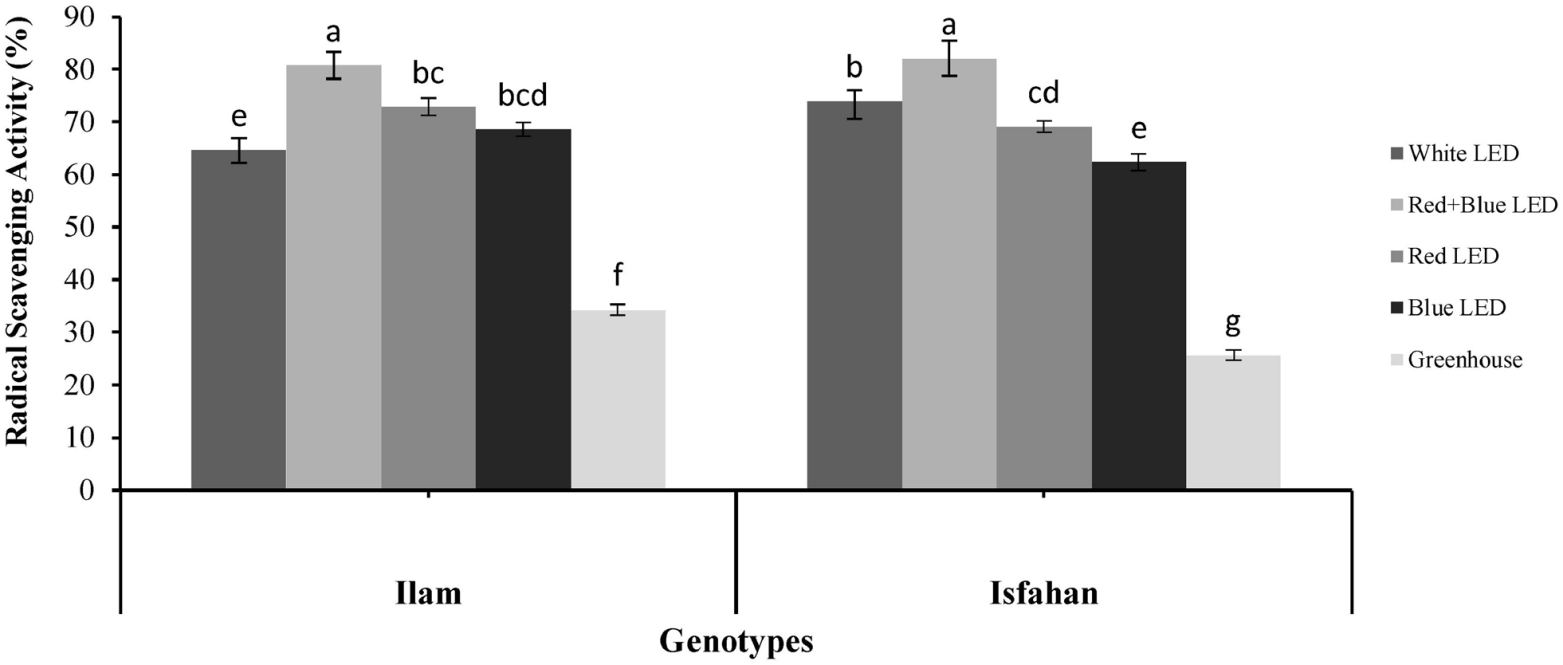
Interaction of genotype*light on free radical scavenging activity in leaves of two genotypes of *Melissa officinalis* affected by different levels of light (non-identical letters indicate significant difference at 0.01 probability level of LSD test)

Results of the analysis of variance of free radical scavenging activity (RSA or antioxidant activity) showed that there was no significant difference between the two genotypes in terms of this activity, while the effect of different lights on RSA was significant (p ≤ 0.01) (Table 1). In both genotypes, the highest RSA% was obtained for plants grown under red + blue LED light (80.77 and 82.09% for Ilam and Isfahan genotypes, respectively), and the lowest was for plants grown under the greenhouse condition (34.29 and 25.67% for Ilam and Isfahan genotypes, respectively) (Fig. 1).

### Principal component analysis

**Fig. 2:**
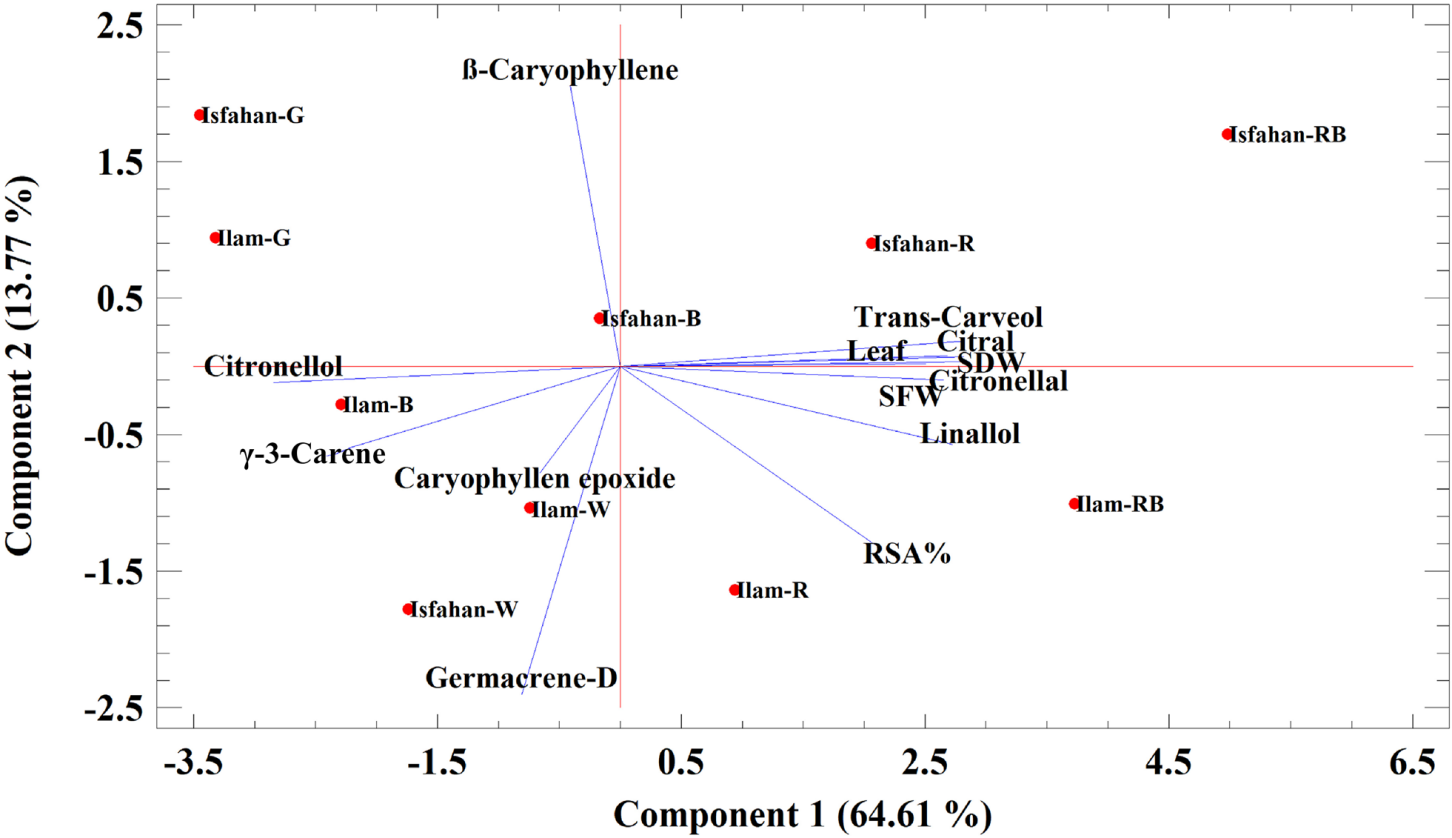
Principal component analysis (PCA) for essential oil components, antioxidant activity, and different wavelengths applied on two genotypes of lemon balm. *Ilam-W* genotype Ilam-White LED, *Ilam-RB* genotype Ilam-Red + Blue LED, *Ilam-R* genotype Ilam-Red LED, *Ilam-B* genotype Ilam-Blue LED, *Ilam-G* genotype Ilam-Greenhouse, *Isfahan-W* genotype Isfahan-White LED, *Isfahan-RB* genotype Isfahan-Red + Blue LED, *Isfahan-R* genotype Isfahan-Red LED, *Isfahan-B* genotype Isfahan-Blue LED, *Isfahan-G*  genotype Isfahan-Greenhouse, *RSA%* radical scavenging activity (%), *SFW* shoot fresh weight, *SDW* shoot dry weight, *Leaf* leaf number

Principal component analysis (PCA) was used to identify the relationship between growth parameters and essential oil components, antioxidant activity, and different wavelengths applied on two genotypes of lemon balm (Fig. 2). The results of PCA analysis identified three main groups and these results also indicated that the first and second groups altogether accounted for 78.39% of the total variation. The first PC (PC1) and the second PC (PC2) revealed 64.61 and 13.77% of the total variation, respectively. The plants grown under LED light sources were completely separated based on the growth parameters, essential oil constituents, and antioxidant activity, from those grown in the greenhouse condition. In terms of caryophyllene, only plants of the Isfahan genotype that were grown in incubators containing red and blue LEDs were similar to greenhouse light. In the other group, Ilam genotype that was under red + blue and red LEDs, and Isfahan genotype grown under red + blue, red and blue LEDs, there were the highest levels of main monoterpene compounds such as citronellal, trans-carveol, linalool, and citral and the highest antioxidant activity plus shoot fresh and dry weights. The third group, the Ilam genotype grown under white and blue LEDs, and Isfahan genotypes with white LEDs also had the same and the highest amount of monoterpene compounds such as citronellol and γ-3-carene and sesquiterpene compounds such as caryophyllene epoxide and germacrene-D.

## Discussion

### The effect of different light sources on growth parameters

In the present study, under red + blue LEDs, the highest values of growth parameters were observed. An increase in fresh and dry weights, leaf area, and chlorophyll content in lettuce plants in studies conducted by Kim et al. (2004), Johkan et al. (2010), and Yorio et al. (2001) showed that the highest amount of these indicators can be seen under the combination of red and blue lights. Also, the combination of red + blue lights increased fresh and dry weights by 3 times compared to the combination of blue and far-red light. It seemed that the combination of red and blue lights increased plant growth by increasing the amount of pure photosynthesis because the red + blue light energy distribution corresponded to the absorption spectrum of chlorophyll (Wang et al. 2007). On the other hand, the intensity of photons from LED lamps is higher than that of other light sources, which is another mechanism of the positive effect of LED lights to improve plant growth indices (Yorio et al. 2001).

### The effect of different light sources on essential oil content and constituents

It is very important to study the change in the amount of essential oil of medicinal plants because it is related to the change in their nutritional, medicinal, and antioxidant properties. Scientists believed that the chemical composition of essential oil and production of secondary metabolites are related to the physiology of the plant, developmental stage, plant species, and various treatments such as various light sources (Yu et al. 2005; Fernandes et al. 2013; Mashkani et al. 2018). Content and composition of plant’s essential oil are influenced by many factors, including environmental conditions (Fernandes et al. 2013; İzgı et al. 2017), light period, light intensity (Shafiee-Hajiabad et al. 2016), and light spectrum (Li and Kubota 2009). The light spectrum acts a significant function in controlling morphology, growth processes, and photosynthesis activities in plants (Wang et al. 2016). Besides, most plant species can promote acclimation systems to cope with different lights, including changing their content of essential oil, which could be one of the mechanisms that plants respond to stress (Zhang et al. 2003). Ivanitskikh and Tarakanov (2014) stated that changes in the light spectrum can be utilized for the biosynthesis of material in plants including essential oils. The light intensity can also alter essential oil production by the stimulation of photo-sensitive enzymes required for the mevalonic acid pathway. Thus, irradiance can directly affect the generation of essential oils, or indirectly, through the increase in plant biomass (Pegoraro et al. 2010). Increasing the number of leaves and trichomes (Fernandes et al. 2013), the effect of light on the expression of genes affecting essential oil synthesis (Urbonavičiūtė et al. 2008), and increasing the number of intermediates involved in essential oil biosynthesis such as IPP (Selmar and Kirinwächter 2013) are some other mechanisms that show the role of natural light in increasing essential oils. Recently, there is a global trend in arid regions of the world including Iran to use greenhouses and indoor facilities instead of the field for growing medicinal species due to the shortage in irrigation water (Mesgaran and Azadi, 2018). In these conditions, LEDs can be used as a supplementary light or sole source of light for boosting photosynthesis and increasing metabolites accumulation. As Darko et al. (2014) stated, LED lights can mimic the effects of natural light, and therefore, it is not unreasonable to expect that LED lights can also increase these compounds by mechanisms similar to what is interpreted about the effect of natural light on increasing the amount of essential oils in plants.

In the study by Batista et al. (2016) on three chemotypes of *Lippia alba*, they demonstrated that the differences in the composition of essential oil were more influenced by the chemotype than light treatments (LED and fluorescent) applied. They and Viccini et al. (2014) stated that this difference between genotypes and chemotypes could be depended on the content of plant DNA.

The content and composition of essential oils in two genotypes studied in the present work were variable under different light treatments and the total amount of compounds in the Ilam genotype was higher than that in the other genotype. However, the observed variation between two genotypes in the essentials oil composition was related to the relative percentage of the constituents and not to the appearance or lack of a specific component. Light quality and its intensity influenced the production of secondary metabolites and essential oils with changes in physiological and morphological properties of plants (Briskin et al. 2001). Light affects the number and morphology of leaves and essential oil storage structures such as trichrome, causing changes in the amount and chemical composition of essential oil in plants (Fernandes et al. 2013).

The results of this study tend to indicate that LED lights have a positive effect on the essential oil content. In the present study, in plants of the Ilam genotype, all LED lights had a greater effect on essential oil content than plants grown under greenhouse conditions but in the Isfahan genotype, only the effect of red + blue and red LEDs treatments was higher than greenhouse conditions. Treatments of red and red + blue LEDs on Ilam and Isfahan genotypes resulted in the greatest essential oil contents. Other studies have suggested the positive effect of LED light on increasing the essential oil content in *Mentha* species, green vegetables (such as parsley, onion, lettuce, and…), and *Brassica oleracea* (Sabzalian et al. 2014; Žukauskas et al. 2011; Lefsrud et al. 2008).

### The effect of different light sources on monoterpenes

The highest amount of monoterpenes in both genotypes were observed under red + blue LEDs. citronellal, trans-carveol, linalool, and citral had the highest levels of monoterpenes in both genotypes. The chemical composition of lemon balm essential oil (that is 0.02–0.3% of DW) had been previously investigated. The main combination was citronellal (2–40%) and citral (10–30%) with ß-caryophyllene, germacrene-D, ocimene, and citronellol (Schultze et al. 1989; Adzet et al. 1992; Kreis and Mosandl 1994; Moradkhani et al. 2010). Citronellal, as one of the major components in the essential oil of lemon balm, is a monoterpene aldehyde (Chung et al. 2010). In the present study, citronellal percentage varied from 10.2 to 17.2%, and the highest amount (17.2%) was detected in plants of both genotypes grown in incubators containing red + blue LEDs. In many studies, citral after citronellal was the most commonly reported composition of lemon balm essential oil, but as was seen in the present study, the highest essential oil content after citronellal was citronellol with a varied range of 6.5–11.8%. In the present study, citral after citronellal, citronellol, and trans-carveol had the highest amount of essential oil in two genotypes under different light treatments.

Light quality can change the composition of essential oils in medicinal plants (Amaki et al. 2011). In a study by Batista et al. (2016), different qualities of light caused a change in the pattern of essential oil in *Lippia alba*. These researchers showed changes in the number of monoterpenes such as eucalyptol and linalool in two *L. alba* chemotypes under LED and fluorescent lamps. Noguchi and Amaki (2016) and Nguyen and Saleh (2019) reported an increase in monoterpenes such as alpha-pinene, beta-pinene, limonene and carvone in mint plants under red LED light.

According to the findings of the present study, it was found that in the two genotypes of lemon balm, light treatments play an effective role in changing the content of monoterpenes. Since most of the essential oil components of lemon balm in this study were monoterpenes, it is concluded that the quality and property of essential oil will probably change significantly. Ghaffari et al. (2019) and Tohidi et al. (2019) reached the same results with the current study about the increase in the content of monoterpenes under LED lamps. In the present study, red + blue LED light was the major contributor to the increase in monoterpenes. On the contrary, in studies by Ghaffari et al. (2019) and Tohidi et al. (2019), blue light played this role. Therefore, this difference reflects the different effects of LED light on the essential oil of different plants.

It is well known that the increase in the production of secondary metabolites is related to plant growth conditions such as temperature, light regime, stress, nutrition source, etc. (Selmar and Kirinwächter 2013). If the light energy that is absorbed by the photosynthetic apparatus exceeds the energy required for CO_2_ fixation, large amounts of this energy ultimately led to the production of secondary metabolites according to the energy dissipation mechanism proposed by Selmar and Kirinwächter (2013). In the present study, since the intensity of light in LEDs (300 μmol.m^−2^.s^−1^) is continuously higher than greenhouse conditions, these light sources, especially red + blue LED light, can be considered as severe light stress and probably increased monoterpenes according to the mechanism described above. Recently, researchers have found that terpenoids biosynthesis in addition to the mevalonic acid (MVA) pathway is made from primary metabolites through another pathway called methylerythritol phosphate (MEP), which is present in plastids and chloroplasts (Lichtenthaler et al. 1999). Therefore, it can be stated that under the treatment of LED lights, especially under red + blue LED light, due to the matching of these wavelengths to the absorption peak of photosynthetic pigments, LEDs increased photosynthesis and production of photosynthetic intermediates, especially IPP (isopentenyl diphosphate). This process may have resulted in the activation of the MEP pathway more and more and produced high levels of these terpenoids.

### The effect of different light sources on sesquiterpenes

Different light sources changed the content of sesquiterpenes of two genotypes of lemon balm. As in Jalal et al.'s study (2015), in this study, the main sesquiterpene of lemon balm was ß-caryophyllene epoxide (11%) that its vector was correlated with white LED light environment in both genotypes (Fig. 2). In the study of Kittler et al. (2018) ß-caryophyllene, germacrene-D, and ß-caryophyllene epoxide constituted the main sesquiterpene compounds of lemon balm genotypes. However, in the study conducted by Chung et al. (2010), caryophyllene (0.8%) and farnesene (0.1%) were the most common sesquiterpene. So, it can be said that the essential oil composition of species and genotypes of a plant are different under different conditions. ß-caryophyllene is a natural bicyclic sesquiterpene that has also been involved in the creation of a spicy taste of black pepper and many essential oils such as *Rosmarinus officinalis*, *Syzygium aromaticum*, and *Cannabis sativa*. This compound has received much attention because of its cyclobutane ring, which is scarce in nature (Taherpour et al. 2012).

Many studies have shown that LED lights induced several metabolic changes in plants, including increased levels of β-farnesene, germacrene-D, and elements in Mexican mint grown under blue light (Noguchi and Amaki 2016). Alvarenga et al. (2015) showed that high light intensities, such as 47 and 69 μmol.m^−2^.s^−1^ increased monoterpene compounds content in *Achillea millefolium* L., but these intensities reduced the number of sesquiterpenes. Sesquiterpenes such as ß-caryophyllene and ß-cubebene have the highest values ​​at 13 μmol.m^−2^.s^−1^ intensity. Blue LED light in *Mentha spicata* increased ß-caryophyllene compared to the control condition. The concentration of beta-bourbonene as a sesquiterpene was also higher in mint cultivated under LED light compared to the control condition (Nguyen and Saleh 2019). An increase in trans-caryophyllene and α-humulene in two species of *Perovskia* has also been reported under the white LED light (Ghafari et al. 2019). Tohidi et al.'s (2019) study showed an increase in sesquiterpene levels of three species of thyme under red + blue and red LED lights.

### The effect of different light sources on radical scavenging activity

In the present work, red + blue LEDs and then red LED had the greatest effect on increasing RSA in plants of two genotypes of lemon balm particularly in the Ilam genotype (Fig. 2). Synthesis of secondary metabolites in plants is an important part of the defense response to stress. Oxidative stress and reactive oxygen species (ROS) cause extensive damages to plants. Antioxidant systems that show antioxidant capacity in plants including flavonoids, ascorbate, carotenoids, phenolic compounds, and essential oils, protect plants against photo-oxidative damage (Shohael et al. 2006).

In general, in addition to genetic differences between different species, it has been found that beside the quality of light, altitude and temperature are important environmental factors that affect plant composition and characteristics (Gharibi et al., 2015). Li and Kubota (2009) demonstrated the beneficial role of LEDs in improving antioxidant activity in baby leaf lettuce. Also, Samuolienė et al. (2012) reported that LED supplementation with HPS lamps altered the antioxidant and nutritional properties of lettuce due to increased metabolic system activity to protect plants against moderate photo-oxidative stress induced by the use of LEDs. Žukauskas et al. (2011) showed that complementary red light had an increasing effect on DPPH removal ability in lettuce, but the antioxidant ability of rice leaves in response to blue LED light was higher than that in red LED light (Jung et al., 2013). Also, in a study by Wojciechowska et al. (2015), 100% red LED exposure did not increase DPPH removal power compared to controls. In the present study, it was observed that the red + blue LED light treatment produced the highest amount of major monoterpene components such as citronellal, trans-carveol, citronellol, linalool, and citral together with the highest RSA% in two genotypes of lemon balm. Therefore, it can be stated that the antioxidant capacity of these two genotypes under light treatments is directly related to the essential oil components, especially monoterpenes. Confirming this result, researchers have shown that DPPH's scavenging activity is directly related to the levels of phenolic and antioxidant compounds of plants and essential oils (Wojciechowska et al. 2015; Tohidi et al. 2017, 2019).

## Conclusion

In the present study, the effect of different sources of LED light and greenhouse light on two genotypes of lemon balm was measured in terms of growth factors, amount and composition of essential oil, and antioxidant activity. Different light sources had a significant effect on the measured characteristics of the two genotypes, which could also alter their medicinal and food properties. In both genotypes, the positive effect of LEDs compared to greenhouse light on the measured properties was quite significant. In both genotypes, red + blue LED produced the highest amount of shoot fresh and dry weights, leaf number, and essential oil monoterpenes, such as citronellal, trans-carveol, linalool, and citral, and the highest amount of antioxidant activity. From the results of the present study, it can be concluded that red + blue LEDs can be introduced as a suitable light treatment for growing and increasing the efficiency of lemon balm plants and probably other medicinal plants. This light treatment has been able to imitate the positive effects of sunlight in increasing the efficiency and performance of lemon balm and has been able to work even better than sunlight. It can be concluded that this light treatment with increasing antioxidant properties could have the best effect on improving the production, and nutritional and pharmaceutical characteristics of lemon balm.

## Supplementary Information

**Additional file 1**: **Fig. S1**. The chromatogram generated from a red light sample of the Ilam genotype.

**Additional file 2**: **Fig. S2**. The chromatogram generated from a white light sample of the Ilam genotype.

**Additional file 3**: **Fig. S3**. The chromatogram generated from a red+Blue light sample of the Isfahan genotype.

**Additional file 4**: **Fig. S4**. The chromatogram generated from a greenhouse light sample of the Isfahan genotype.

## Data Availability

Agree.

## Abbreviations

LED: Light-emitting diode
RSA: Radical Scavenging Activity
DPPH: 2,2-Diphenyl-1-picrylhydrazyl
MEP: Methylerythritol phosphate
IPP: Isopentenyl diphosphate
PCA: Principal Component Analysis
