# Supplementary figures and images for: LED light sources improved the essential oil components and antioxidant activity of two genotypes of lemon balm (Melissa officinalis L.)
Source: Bot Stud. 2021 Jun 5;62:9. doi: 10.1186/s40529-021-00316-7 (PMC8179865; doi:10.1186/s40529-021-00316-7)

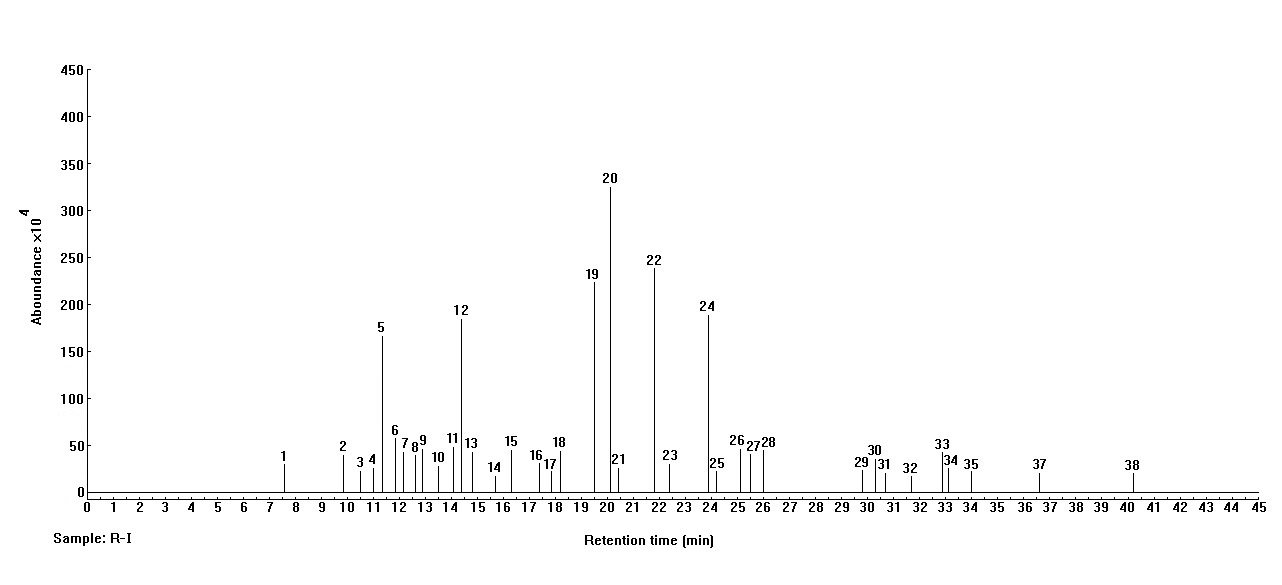

Supplement: Supplementary file 1 — Additional file 1: Fig. S1. The chromatogram generated from a red light sample of the Ilam genotype. [file 40529_2021_316_MOESM1_ESM.jpg]

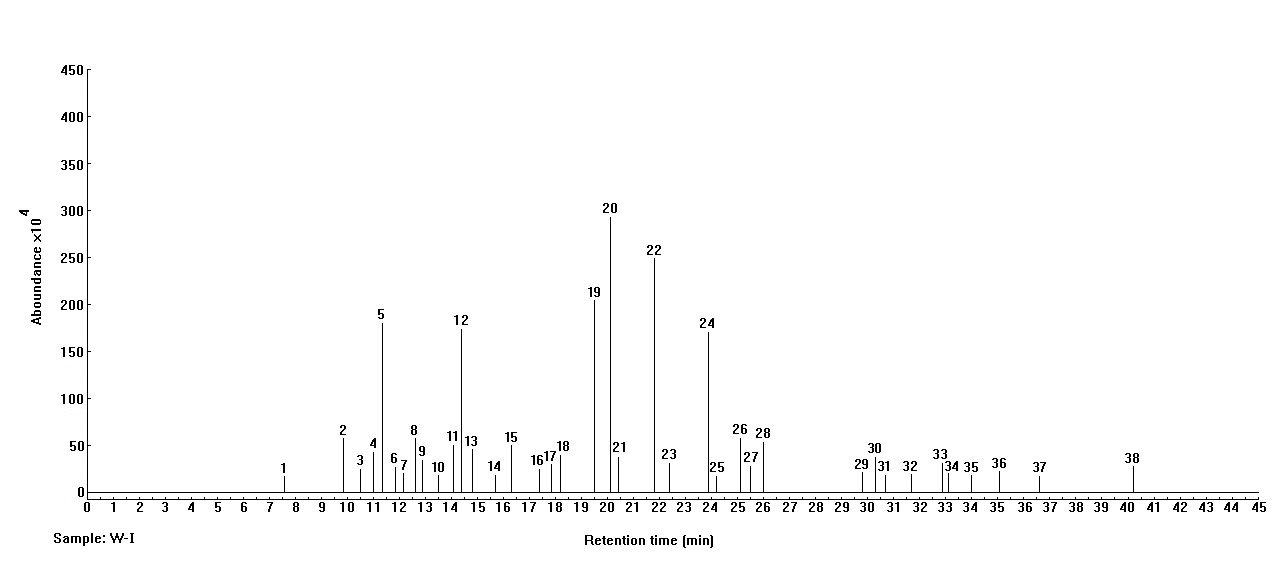

Supplement: Supplementary file 2 — Additional file 2: Fig. S2. The chromatogram generated from a white light sample of the Ilam genotype. [file 40529_2021_316_MOESM2_ESM.jpg]

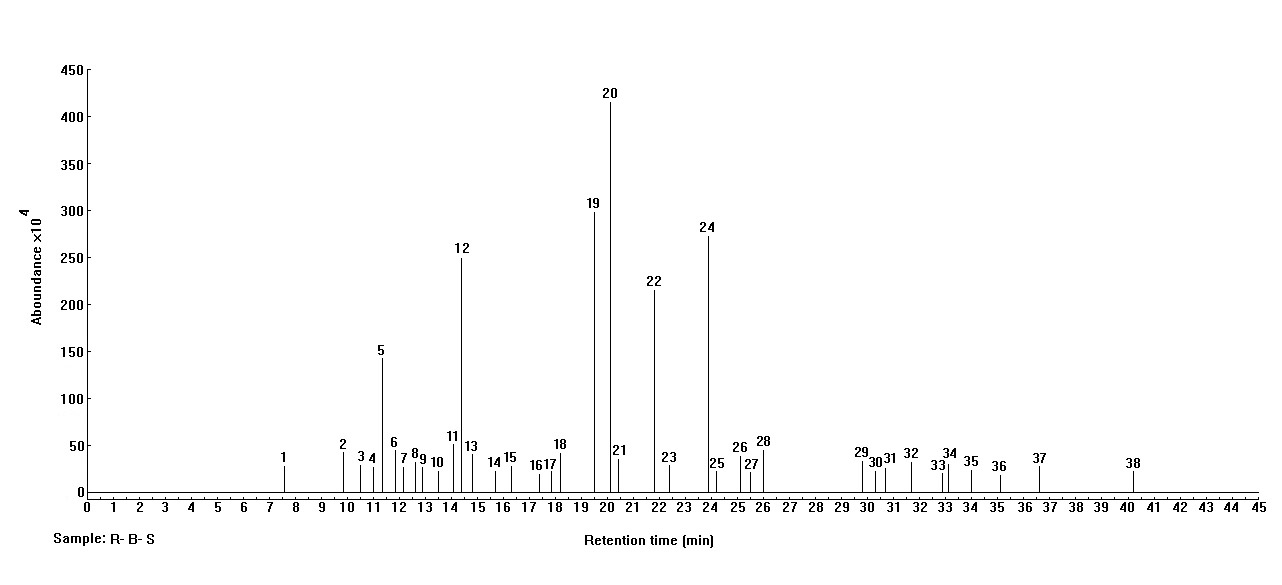

Supplement: Supplementary file 3 — Additional file 3: Fig. S3. The chromatogram generated from a red+Blue light sample of the Isfahan genotype. [file 40529_2021_316_MOESM3_ESM.jpg]

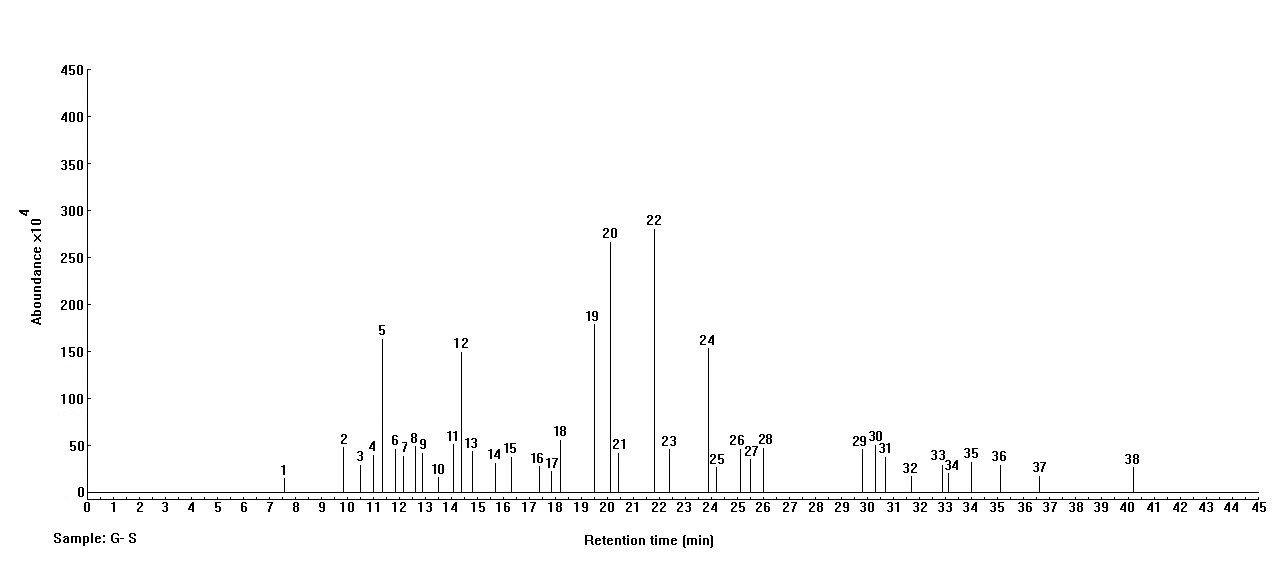

Supplement: Supplementary file 4 — Additional file 4: Fig. S4. The chromatogram generated from a greenhouse light sample of the Isfahan genotype. [file 40529_2021_316_MOESM4_ESM.jpg]
